# Determinants of delayed specialist presentation in head and neck cancer to Tygerberg Hospital, Western Cape

**DOI:** 10.4102/jcmsa.v3i1.215

**Published:** 2025-09-12

**Authors:** Petrus J. Vermeulen, Johan Grobbelaar

**Affiliations:** 1 Department of Otorhinolaryngology, Faculty of Surgery, Stellenbosch University, Cape Town, South Africa

**Keywords:** barriers, head and neck cancer, referral, delayed presentation, advanced disease

## Abstract

**Background:**

Delayed presentation of head and neck cancers at Tygerberg Hospital often results in tumour progression and clinical upstaging – frequently marking the difference between curative and palliative care. This study aimed to identify factors contributing to prolonged time intervals before specialist consultation in South Africa’s public healthcare sector, and to inform potential interventions to address these delays.

**Methods:**

A cross-sectional, survey-based study was conducted. Patients were recruited weekly, and data were collected through individual interviews. Descriptive statistics were used to analyse responses.

**Results:**

At initial specialist presentation, 58.9% of patients were diagnosed with stage III/IV disease, with oral cavity malignancies being most common. Nearly half (47.06%) experienced delays because of inappropriate antibiotic prescriptions and misdiagnoses. The median time from symptom onset to specialist review was 135 days. The most frequently cited patient-related barrier was poor health literacy (57.84%).

**Conclusion:**

The majority of patients presented with advanced-stage head and neck cancer. Delays were attributed to both clinician-related factors – such as misdiagnosis and unwarranted antibiotic use – and patient-related factors, primarily limited health literacy. The observed median delay exceeds international benchmarks, underscoring the need for targeted interventions.

**Contribution:**

This study highlights the urgent need for earlier referral and specialist assessment of head and neck cancer patients. Findings support the implementation of clinician education, patient awareness initiatives and policy development aimed at reducing diagnostic and referral delays within South Africa’s public healthcare system.

## Introduction

The South African public healthcare system is characterised by a largely under-resourced sector serving the majority of the country’s population.^1^ Numerous studies have described an array of factors that influence access to care in the public healthcare system. Past and current socio-economic inequities have displayed the discrepancies experienced by the population in terms of their access to healthcare.^1,2^

It is well established that South African patients experience barriers in accessing cancer care in the public healthcare system. Numerous studies have been conducted to investigate the several reasons for patients who present late and with advanced stages of the disease. Studies, focusing particularly on breast, cervical and prostate cancer, have concluded that poor health literacy, socio-cultural responsibilities (family role, religion, etc.), geographical limitations, lack of transportation, financial burden, low education level as well as the fear of cancer diagnosis or stigma linked to certain cancers are all relevant to delayed presentation of cancer patients.^3,4,5,6^ Delays in presentation occur across both low- and high-income countries. A variety of factors have been identified as the causes of delayed presentation in patients with head and neck cancer, including lack of knowledge, distance to healthcare facilities, low education level and traditional–cultural healthcare beliefs in countries with a similar socio-economic composition as South Africa, but it is nevertheless still poorly studied.^7^

Head and neck surgeons at Tygerberg Hospital observed the consequences of delayed presentation in patients with head and neck cancers, including tumour progression and clinical upstaging of the disease. On the African continent, it has been estimated that up to 83% of patients present in advanced stages of the disease, stage III or IV, requiring complex resection procedures and multimodal treatment plans.^8,9,10^ Unpublished in-house audits from 2023 confirmed that more than 85% of patients present with stage III or IV of the disease. Imaging studies have also concluded that a 30-day delay is associated with a 62% increase in the tumour size, 20% new nodal involvement as well as clinical tumour, node and metastasis (TNM)-upstaging in 16% of the studied population waiting for chemotherapy after a diagnosis was made.^11^ It is for this reason that establishing the determinants of an increased time interval to presentation is crucial as a relatively brief period of 30 days, as seen in the study mentioned above, can result in such adverse consequences. It is for this reason that this study is defining an increased time interval to specialist presentation as a period of more than 30 days between onsets of symptoms and presentation to a specialist. Similar measured time intervals have been used in other countries in different studies that have been comparable with the present study.^12^

The ‘Three Delays Framework’ uses: (1) seeking care; (2) reaching care; and (3) receiving care as a description of stages in which patients can experience delays in accessing care.^10,13^ It is presumed that the reasons for an increased time interval to presentation in patients within our study population arise within the first stage of this framework, namely seeking care. Despite the detrimental impact of patients experiencing an increased time interval to specialist presentation, no studies exist with regard to the determinants of an increased time-interval to presentation in head and neck cancer patients in the South African context.

The primary aim of this study is to identify the determinants of an increased time interval to specialist presentation in patients with head and neck cancer in the public healthcare sector in South Africa. The study also secondarily aimed to provide literature for the development of interventions directed at reducing time-intervals to presentation, both to primary care and specialist level, in patients with head and neck cancer in the public healthcare sector in South Africa.

The objectives of the study are to ascertain patients’ perspectives on the factors contributing to increased time intervals before presentation, to examine the impact of socio-economic factors on these delays and to determine whether the referral process itself contributed to prolonged time intervals. Furthermore, the study aims to record the prevalence of various TNM stages at presentation within the study population and to describe the frequency of late presentations to specialist care in the cohort.

## Research methods and design

### Study design

The study design is a cross-sectional study, formatted in a survey.

### Setting

The study’s setting was within the Division of Otorhinolaryngology, Department of Surgery, Faculty of Medicine and Health Sciences, Stellenbosch University and Tygerberg Academic Hospital. The hospital forms part of South Africa’s public healthcare system and provides services to patients from both urban and rural areas, mostly from lower socio-economic background. It is also one of the two public tertiary hospitals in South Africa that treat patients with head and neck cancer. This hospital serves a drainage area of approximately 3.8 million people. It is the second-largest hospital in South Africa, and annually on average 107 215 patients are admitted, with 492 670 seen at outpatient clinics and 30 784 operations performed. The hospital accommodates daily average of approximately 10 000 inpatients and outpatients. The Division of Otorhinolaryngology sees, on average, 30 patients with newly diagnosed head and neck cancers each month.^14^

### Study population and sampling strategy

Participants included in the study were all patients attending the head and neck cancer and oncology clinic in the Division of Otorhinolaryngology at Tygerberg Hospital with a histopathological diagnosis of head and neck cancer. Excluded from participation were patients who did not give consent to participate in the study, and also all the patients with a diagnosis other than a cancer of the head and neck and children (aged 0–17 years).

When looking at similar studies conducted in other population contexts, study sample sizes varied. A sample range of 39–173 patients was used in studies using a similar time period for data collection (< 1 year).^7,15,16,17^ It is for this reason that a sample size of 100 patients, presenting 30 days after onset of symptoms, was sufficient for a conclusion. None of the patients who attended the head and neck cancer clinic was excluded from participation.

### Data collection

The method used for data collection was a survey created on the electronic database website, REDCap. The study and survey were explained to the patients, and consents were obtained to participate in the study, whereafter the survey was completed by the principal investigator, P.V., during a patient interview in a private room. Data were collected from 01 April 2024 to 04 December 2024.

The study collected data on several key variables, including the date of symptom onset, the date of the patient’s initial presentation to a healthcare worker and the date of their first visit to an otorhinolaryngology specialist. It also recorded the definitive diagnosis using the International Classification of Diseases (ICD-10) codes and the stage of cancer at initial diagnosis, according to the 8th edition of the TNM-staging system. Additionally, the study documented any previous misdiagnoses or treatments received for the condition, identified the referring healthcare professional and gathered various patient-specific details such as education level, combined monthly household income, property ownership and any prior history of cancer. Finally, the study explored the patients’ perspective on barriers to accessing healthcare for their condition.

Information relating to the patient’s diagnosis and cancer staging was obtained from the patient’s oncology assessment sheet. For the purpose of the study, only the patient’s ICD-10 diagnosis and the histopathological TNM-staging were recorded. This information was also collected through the same REDCap survey, without including any identifiable information of the study participants.

### Data analysis

Once the data had been collected onto REDCap through the individual surveys, the data were compiled into an Excel spreadsheet. Through this format, statistical analysis was conducted by using the statistical analysis software, SPSS (version 29.0).

Data were descriptively analysed in this format and presented through standard statistical analytical methods. Categorical variables were calculated in frequencies and proportions, while continuous variables were calculated in mean and standard deviation, or median and interquartile ranges. Proportion estimates were reported with a 95% confidence interval (CI) for patient specialist presentation ranges. Variables and measured presentation outcomes were compiled into a linear regression model to determine significant association, also with CIs reported at 95% and a *p*-value of < 0.05 deemed as statistically significant.

### Ethical considerations

Ethical clearance to conduct this study was obtained from Stellenbosch University, Undergraduate Research Ethics Committee on 09 February 2024 (HREC Reference No: U23/10/296). Approval was also obtained from Tygerberg Hospital and the study was registered with the Provincial Department of Health. All procedures performed in the studies involving human participants were in accordance with the ethical standards of the institutional and/or national research committee, with the 1964 Helsinki Declaration, and its later amendments or comparable ethical standards. Written informed consent was obtained from all individual participants involved in this study. Data were entered in a de-identified manner and therefore, no information of the participants will be available to identify patients.

## Results

A total of 102 patients participated in the study. Table 1 contains the TNM-staging of the participants, whilst Table 2 presents the stage of the head and neck cancer upon specialist presentation.

**TABLE 1:** Tumour, node and metastasis-staging of diagnosis of study participants at specialist presentation.

Staging	Frequency	%
T1	6	5.88
T2	19	18.63
T3	44	43.13
T4a	30	29.41
T4b	3	2.94
N0	41	40.20
N1	14	13.73
N2a	7	6.86
N2b	9	8.82
N2c	11	10.78
N3a	6	5.88
N3b	14	13.73
M0	96	94.12
M1	6	5.88

**TABLE 2:** Stage of participants upon specialist presentation.

Stage	Frequency	%
0	0	0.00
I	4	3.92
II	8	7.84
III	28	27.45
IVa	39	38.24
IVb	17	16.67
IVc	6	5.88

Referrals to Tygerberg Hospital were 86 (84.31%) patients by general practitioners or medical officers, 7 patients (6.86%) by a dentist, 4 patients (3.92%) by maxillofacial surgeons, 2 patients (1.96%) by general surgeons, 2 patients (1.96%) by other otorhinolaryngologists and 1 (0.89%) patient by a nurse practitioner.

Out of the 102 patients, 52 (50.98%) were not misdiagnosed during initial presentation with symptoms to a healthcare professional. A total of 48 (47.06%) patients were specifically treated with antibiotic therapy upon initial presentation to a healthcare worker and therefore judged to be misdiagnosed. Out of the total patients, 1 (0.89%) patient was diagnosed with gastritis initially and 1 (0.89%) patient was diagnosed with Bell’s palsy upon initial presentation to a healthcare worker.

Socio-economic background of the participants was reported as follows:

- Schooling level was reported as follows: 5 (4.90%) patients had no formal schooling, 25 (24.51%) had some primary education, 18 (17.66%) had completed primary education, 38 (37.25%) had some secondary education, 11 (10.78%) did complete secondary education and 5 (4.90%) had a tertiary qualification.
- Twenty (20.59%) patients reported no income, 10 (9.80%) had an income of R0.00–R1600.00, 36 (35.29%) an income of R1601.00–R3200.00, 20 (19.61%) an income of R3201.00–R6400.00, 11 (10.78%) an income of R6401.00–R12 800.00, 2 (1.96%) an income of R12 801.00–R25 600.00 and 2 (1.96%) had an income of R25 601.00–R51 200.00.
- Twenty-nine (28.43%) of the patients owned property, while the remaining 73 (71.5%) patients did not own any property.
- Eight (7.84%) of the participants had a previous history of a cancer diagnosis. Ninty-four (92.16%) of the participants had never been diagnosed with cancer before the current presentation.

The most common primary sites of tumours were floor of mouth followed by base of tongue. Breakdowns of all primary sites according to ICD-10 codes are presented in Table 3.

**TABLE 3:** Primary sites (according to ICD-10 codes).

ICD-10 code	Histological diagnosis	Frequency	%
C01	Malignant neoplasm of base of tongue	9	8.82
C02.1	Malignant neoplasm of border of tongue	4	3.92
C02.3	Malignant neoplasm of anterior two-thirds of tongue	1	0.98
C02.9	Malignant neoplasm of tongue, unspecified	6	5.88
C03.1	Malignant neoplasm of the lower gum	1	0.98
C03.9	Malignant neoplasm of the gum, unspecified	2	1.96
C04.9	Malignant neoplasm of the floor of the mouth	12	11.76
C04.1	Malignant neoplasm of the lateral floor of the mouth	3	2.94
C05.9	Malignant neoplasm of the palate, unspecified	2	1.96
C05.1	Malignant neoplasm of the soft palate	2	1.96
C06.2	Malignant neoplasm of retromolar area	4	3.92
C06.9	Malignant neoplasm of the mouth, unspecified	4	3.92
C07	Malignant neoplasm of the parotid gland	5	4.90
C09	Malignant neoplasm of the tonsil	5	4.90
C10.1	Malignant neoplasm of the anterior surface of the epiglottis	3	2.94
C10.2	Malignant neoplasm of the lateral wall of the oropharynx	2	1.96
C10.9	Malignant neoplasm of the oropharynx, unspecified	6	5.88
C11.8	Malignant neoplasm overlapping lesion of the nasopharynx	1	0.98
C11.9	Malignant neoplasm of the nasopharynx, unspecified	1	0.98
C13.2	Malignant neoplasm of the posterior wall of the hypopharynx	1	0.98
C13.9	Malignant neoplasm of the hypopharynx, unspecified	3	2.94
C14.0	Malignant neoplasm of the pharynx, unspecified	2	1.96
C14.9	Malignant neoplasm overlapping lesion of the lip, oral cavity and pharynx	2	1.96
C31.0	Malignant neoplasm of the maxillary sinus	1	0.98
C31.1	Malignant neoplasm of the ethmoidal sinus	1	0.98
C31.3	Malignant neoplasm of the sphenoidal sinus	1	0.98
C32.0	Malignant neoplasm of the glottis	2	1.96
C32.1	Malignant neoplasm of the supraglottis	6	5.88
C32.9	Malignant neoplasm of the larynx, unspecified	1	0.98
C41.1	Malignant neoplasm of the mandible	2	1.96
C44.2	Malignant neoplasm of the skin of the ear and external auricular canal	1	0.98
C77.0	Secondary and unspecified malignant neoplasm of the lymph nodes of the head, face and neck	2	1.96

Time intervals were categorised into three different intervals: (1) onset of symptoms to healthcare worker presentation; (2) healthcare worker to specialist presentation; and (3) onset of symptoms to specialist presentation. The time intervals recorded from patient interviews are reported in both Table 4 and Table 5.

**TABLE 4:** Categorical time intervals for presentation of study participants.

Time-interval	25th interquartile range (days)	Median (days)	75th interquartile range (days)
Onset of symptoms to healthcare worker presentation	23.00	49.00	104.00
Initial healthcare worker to specialist presentation	22.00	64.00	120.00
Onset of symptoms to specialist presentation	68.00	135.00	260.00

**TABLE 5:** Frequency of participants that presented within 30 days of onset of symptoms to specialist care.

Time interval from onset of symptoms to specialist presentation	Frequency	%	95% CI
Less than/equal to 30 days (≤ 30 days)	2	2.00	0.05% – 6.90%
Greater than 30 days (> 30 days)	100	98.04	-

CI, confidence intervals.

Participants who were treated with antibiotics prior to a specialist referral took a median of 54.5 days longer to reach specialist care after initial contact with a healthcare worker (95% CI: 27–82 days; *p* < 0.001). Table 6 presents the time intervals, measured in median days, for study participants who were prescribed antibiotics *versus* participants without antibiotic prescription. Referral from a general practitioner, income level, property ownership and education level did not contribute as statistically significant determinants of delayed presentation to specialist care following consultation with a healthcare worker. Patients with metastasis who present at specialist presentation did not statistically significantly contribute to late presentation from a prior visit to a healthcare professional. No significant determinants were reported that contributed to delay from onset of symptoms to specialist presentation as well as onset of symptoms to presentation to a healthcare worker, including antibiotics prescription, referral from a general practitioner, income level, property ownership, education level or the presence of metastasis.

**TABLE 6:** Comparative analysis of categorical time intervals in patients with and without antibiotic prescription before specialist presentation.

Antibiotic prescription status	Time-interval	25th interquartile range (days)	Median (days)	75th interquartile range (days)
Without antibiotics prescribed	Onset of symptoms to healthcare worker presentation	36.00	57.00	181.00
	Initial healthcare worker to specialist presentation	9.00	25.50	85.00
	Onset of symptoms to specialist presentation	61.00	112.00	287.00
With antibiotics prescribed	Onset of symptoms to healthcare worker presentation	11.50	33.00	84.50
	Initial healthcare worker to specialist presentation	56.50	83.50	128.00
	Onset of symptoms to specialist presentation	79.50	162.00	252.50

Barriers to presentation, as identified by participants in the study, are presented in Table 7. These barriers were identified after personal interviews conducted with participants after they were informed of their cancer diagnosis revealed in the head and neck cancer clinic.

**TABLE 7:** Barriers identified by participants to presentation.

Barriers to presentation	Frequency	%
Poor health literacy	59	57.84
Conflicting or misleading information from healthcare professionals	45	44.12
Poor care received with regards to complaint at local Clinic and/or hospital	28	27.45
Tumour characteristics	18	17.65
Access to transport	14	13.73
Use of traditional medicine	6	5.88
Unable to leave household responsibilities	5	4.90
Substance use	3	2.94
Inability to receive paid leave from employer	1	0.98
Fear of the doctor and/or hospital	0	0.00
Other: Scared of receiving a cancer diagnosis	2	1.96
Other: Problems relating to medical aid	1	0.98

*Poor health literacy* was reported after questioning patients about their knowledge on their condition such as the risk factors associated, warning signs of cancers as well as the disease process and prognosis at the stage of diagnosis. *Misleading or conflicting information* from various healthcare professionals included wrongful referrals, diagnoses and treatment from clinicians. *Poor care received with regard to their complaint at the local clinic and/or hospital* was defined as an obstruction to receiving care at clinic and/or hospital (e.g. long waiting times, together with not being seen at the end of their visit to clinic and/or hospital) as well as not receiving an escalation of treatment during a subsequent visit to the same healthcare facility (often multiple times). *A tumour characteristic* was defined as ‘any factor of a cancerous lesion that did not contribute to alerting the patients of any danger’. This included factors such as slow growth rates, alleviation of symptoms through medication, as well as pre-formed concepts about their lesions. Only 3 out of the 102 participants felt that the options provided were not sufficient for them. Two (*n* = 2) of the patients (1.96%) indicated that being afraid of a cancer diagnosis was a barrier to them presenting to a healthcare professional. The other remaining patient (0.98%) indicated that medical aid issues were the reason for them experiencing delay in reaching specialist care.

## Discussion

This study aimed to describe the pre-diagnostic barriers that patients with head and neck cancer experienced, leading to delayed presentation. We are aware that late presentation leads to increases in tumour stage and is accompanied by a decline in disease prognosis.^16^ Our study population demonstrated that 75.48% presented with a tumour stage (T-stage) of T3, T4a or T4b. In terms of nodal involvement, 59.80% of the participants presented with a nodal stage (N-stage) of N1–N3b. This supports our hypothesis that the majority (88.24%) of our head and neck cancer patients present with advanced stages of disease (stage III and IV).

Primary site of the tumours upon specialist presentation was mostly of the oral cavity, with a malignant neoplasm of the floor of the mouth being the commonest diagnosis. This is surprising as one would expect that a lesion of the oral cavity would lead to earlier patient presentation because of the irritative effect of a lesion in the mouth. However, it is understandable that an oral neoplasm can lead to diagnostic uncertainty with a benign oral lesion or ulcer being morphologically similar in early initial presentation.

Upon initial presentation to a healthcare worker, about half of the patients were correctly diagnosed with a suspicion of a head and neck cancer. Of the remaining group of participants, the majority (47.06%) were subjected to uncertain diagnosis and either single or multiple antibiotic courses (after subsequent visits to the same facility), with the hope that it would resolve the lesion present. One can appreciate that diagnostic uncertainty and lack of clinician knowledge play a role in delayed presentation to specialist care. General practitioners made up 86 (84.31%) out of the 102 referrals to the hospital. This was expected as primary care level is the highest source of referrals to a tertiary care institute.

With regards to time intervals to presentation, it took a median of 49 days for a patient to present to a healthcare worker after the onset of their symptoms. The interval from initial presentation to specialist presentation was a median of 64 days. It was reported that the time interval for patients from onset of symptoms to specialist presentation was a median of 135 days. Only two (1.96%) patients presented to specialist care level within 30 days of onset of symptoms. One hundred (98.04%) patients presented to specialist care after more than 30 days since the onset of symptoms (95% CI: 0.05% – 6.9%). Study participants who were treated with antibiotics prior to specialist referral took a median of 54.5 days longer to reach specialist care after contact with a healthcare worker (95% CI: 27–82 days; *p* < 0.001). This is a statistically significant determinant of an increased time-interval to specialist presentation and supports our hypothesis that diagnostic uncertainty and lack of clinician knowledge at primary care level contribute to poorer patient outcomes and prognosis upon specialist presentation.

When it comes to the obstructions identified by the participants themselves to earlier presentation, firstly, it was reported that the majority of the participants (57.84%) had a poor health literacy level. Patients who present to the Tygerberg Hospital in Southern Africa with a head and neck cancer are typically uneducated with regard to risk factors, disease processes and prognosis as well. This was deemed a major contributor to delays in seeking help and subsequent referral to specialists. Extensive counselling plays a significant role in explaining diagnoses to patients with cancer. Secondly, 44.12% of the participants indicated that they could not present earlier to specialist care because of misleading or conflicting information they received after initial presentation to a healthcare worker. This includes misdiagnosis as well as antibiotic prescriptions, which have already been shown to contribute to delays in reaching specialist care. This shows that the healthcare system at primary care level is failing to provide care to cancer patients in a comprehensive and well-informed manner.

The population mostly did not complete their education up to a level of secondary education. Most participants did not own housing or property. Moreover, 65.68% of participants earned a household income level of less than R3200.00 per month, with most participants being dependent on social grants such as a pension grant. This, however, is typically the demographics of patients attending public healthcare institutions in South Africa.

### Strengths and limitations

A limitation of this study is that the data were collected based on patients’ trustworthiness and recollection during interviews, which may introduce recall bias.

It would have been useful to determine the number of healthcare visits before specialist presentation. This could illustrate even further the scale of intervention needed to reduce time to specialist presentation, as this not only burdens the patient but also the healthcare system.

### Implications or recommendations

Further research on the referring side of the patient’s care would be beneficial in determining barriers to earlier referral from a referring clinician’s perspective.

The findings of this study highlight the importance of both patient and clinician health educational needs. A lack of knowledge or health literacy of head and neck cancers contributes to delayed presentation to specialist care. Primary care clinicians need to be educated on the warning signs of head and neck cancers and patients should be informed about when to seek medical attention if a lesion arises. Educational posters in primary care facility waiting areas could help achieve this. Clinician education can take place in the form of governmentally endorsed continued professional development (CPD) webinars or educational articles, mandatory for all practising clinicians. Through providing educational material about the diagnosis and management of head and neck cancer, clinicians can be upskilled in their practice, while also earning mandatory CPD points annually. Nursing education can occur through amending the Adult Primary Care (APC) guideline or Practical Approach to Care Kit (PACK) guidelines that are revised often, as little to no information with regards to head and neck cancer is included in this guideline to date. This guideline is available in all primary care facilities as a structured approach to different diseases and the management thereof, and can be easily accessed online. This is pertinent as there was an apparent lack of understanding and stewardship displayed with regard to antibiotic prescription during the study in patients presenting with head and neck cancer. Patient and clinician education should be of immense importance for the Department of Health on Provincial and National level, as this not only will improves morbidity and mortality of patients, but reduce the cost of patient care in an already resource-stricken healthcare system (through minimising the need for complex oncological surgeries).

Furthermore, Otorhinolaryngological departments should aim to capture data with regard to the referral pathways of patients diagnosed with a head and neck cancer, not only as a good internal data capturing principle but also to aid in highlighting the need for earlier referrals.

## Conclusion

This research study describes the barriers head and neck cancer patients experienced from earlier presentation to specialist care. The magnitude of the problem of delayed presentation was displayed by gathering that most patients were diagnosed with advanced stages (III and IV) of head and neck cancer upon specialist examination. The time interval to specialist presentation from onset of symptoms for the study population was calculated at a median of 135 days, which is far more than international standards prescribe as a successful referral pathway system in patients with a head and neck cancer. Clinician factors contributing to the delay in specialist presentation included a lack of clinical knowledge, evident in the frequent misdiagnosis of the disease and the inappropriate prescription of antibiotics for cancerous lesions. During the interviews, we ascertained that poor health literacy was the predominant reason for delay in reaching specialist care from the patient’s perspective, followed by poor care received with regard to their complaint at primary care level. Socio-economic factors did not have a statistically significant effect on the delay in presentation of patients, but the depiction of the study population comprised mostly low-income, unschooled and grant-dependent patients.
